# Network ecology: Tie fitness in social context(s)

**DOI:** 10.1016/j.socnet.2023.09.005

**Published:** 2023-09-29

**Authors:** Malte Doehne, Daniel A. McFarland, James Moody

**Affiliations:** a University of Zurich, Department of Sociology, Andreasstr. 15, CH-8050 Zürich, Switzerland; b Stanford University, USA. CERAS, 94305 Stanford, CA, United States; c Duke University, 268 Soc/Psych Building, 27708 Durham, NC, United States

**Keywords:** Network ecology, Tie fitness, Relational niches, Timescale separation, Relational, Dynamics, Coevolution, Interactions, Network theory

## Abstract

Social relations are embedded in material, cultural, and institutional settings that affect network dynamics and the resulting topologies. For example, romantic entanglements are subject to social and cultural norms, interfirm alliances are constrained by country-specific legislation, and adolescent friendships are conditioned by classroom settings and neighborhood effects. In short, social contexts shape social relations and the networks they give rise to. However, how and when they do so remain to be established. This paper presents network ecology as a general framework for identifying how the proximal environment shapes social networks by focusing interactions and social relations, and how these interactions and relations in turn shape the environment in which social networks form. Tie fitness is introduced as a metric that quantifies how well particular dyadic social relations would align with the setting. Using longitudinal networks collected on two cohorts each in 18 North American schools, i.e., 36 settings, we develop five generalizable observations about the time-varying fitness of adolescent friendship. Across all 252 analyzed networks, tie fitness predicted new tie formation, tie longevity, and tie survival. Dormant fit ties cluster in relational niches, thereby establishing a resource base for social identities competing for increased representation in the relational system.

## Introduction

Studies of social networks reliably identify a limited number of mechanisms that generate social relations across settings and over time. Friendships, for example, are generally reciprocated, they tend to form among individuals who are similar in salient characteristics, and they incline towards closure such that two individuals who are friends with the same third are more likely to become friends (Block, 2018; Goodreau et al., 2009; Lazarsfeld and Merton, 1954; Martin, 2009; Wimmer and Lewis, 2010). There are socially substantive reasons for the consistency with which voluntaristic relations form (Heider, 1946; Gould, 2002; McPherson and Smith-Lovin, 2001), yet a puzzle remains: despite basic conformity to these mechanisms at the micro level, we find widely differing network topologies at the macro level (McFarland et al., 2014). What explains the emergence of different network structures from the same relational mechanisms?

One reason for the observed macro variation is that social networks are embedded in broader cultural, institutional, and material contexts that favor one relational configuration over another. Indeed, many studies have documented the diverse contexts in which social networks form and the resulting diversity in network structures (Adams et al., 2012; Bidart et al., 2020; Entwisle et al., 2007; Mollenhorst et al., 2008; Burt and Batjargal, 2019). Some contexts regulate contact explicitly by defining who may interact with whom for how long (McFarland et al., 2013). Others are more indirect, as when career advancement is tied to membership in dynamically evolving social circles (Lazega et al., 2017), functionaries are rotated in and out of office (Woldense, 2018), and adolescents know intuitively not to date their ex-partner’s new partner’s ex (Bearman et al., 2004) or their friend’s ex-partner (McMillan et al., 2022). In other contexts, connectivity emerges from unstructured activity (Diehl and McFarland, 2012) and happenstance (Browning et al., 2017; Doehne and Rost, 2021). Collectively, these and other studies demonstrate that social contexts shape network dynamics and outcomes. However, they do not explain *how* context matters, only *that* networks differ across contexts and over time.

Here, we propose a two-part ecological explanation for the macro-level variation puzzle. The first part is that network feedback processes in relational mechanisms have multiplier effects that turn intuitively small differences in how micro-mechanisms are enacted into large topological differences. This means that two places might both favor reciprocity more than social hierarchy, but small differences in emphasis result in quite different network topologies. This is a mechanistic result that is well known from the literatures on random graphs (Newman, 2018) and on ERGM degeneracy (Handcock et al., 2003; Hunter et al., 2008; Snijders et al., 2006; Block et al., 2018) that has important substantive effects (McFarland et al., 2014). Unfortunately, investigations of these feedback processes, including our own earlier work, are theoretically underspecified and rely on stable and homogenous micro-mechanisms that, while tractable, are phenomenologically unlikely.

The second part of our explanation is that variation in micro-mechanism emphasis is subject to ecological selection that solidifies differences not only *between* but also *within* settings. The social history of each setting bounds normative network institutions, codified as social identities, which define what counts as acceptable relational behavior in that context. These institutions reduce the vast combinatorial potential that exists in a population in which anyone could in principle connect to everyone else to a more ‘manageable’ subset of viable connections for individuals to consider. Within limits, settings thus develop and exhibit idiosyncratic social identities and relational norms that channel interactions and shape the evolution of social relations. Consequently, variation across settings in average relational practices depends on how variation within settings is dampened and channeled.

In the following sections, we develop basic elements of a network-ecological framework by which to model ecological constraint on variation in relational activity. We draw on parallel developments in ecology and hierarchy theory to model social networks as evolutionary accomplishments: the emergent outcomes of individuals adapting their fleeting interactions to more durable, higher-level configurations of social relations, local configurations, and social identities. This framework highlights ecological pressures operating at distinct timescales as explanations for - and predictors of – social network dynamics. We introduce a ‘fitness’ metric to summarize how well any one dyad (would) align with the relational norms of a setting if activated. Fit but unexpressed dyads are identified as ‘dormant ties’, a resource base that individuals enacting role-differentiated identities can leverage to pursue their idiosyncratic goals. We use a simple simulation approach to introduce diversity in social identities and relational norm heterogeneity into the analysis of social network dynamics. Using longitudinal network data collected in 36 settings (comprising two student cohorts each tracked over time within 18 North American schools), we model, predict, and explain new tie formation, stability, and longevity through this ecological perspective. While we think the basic tenets of the framework will apply to diverse social network contexts across scales, we focus here on interpersonal networks to keep the theory manageable and exact and leave other sorts of aggregate or corporate relations to future extensions.

## Network ecology: a general framework

### Background

Ecological arguments have been put to productive use throughout the social and behavioral sciences, including sociology (Abbott, 2005; Hawley, 1950; Park and Burgess, 1921; Turner and Machalek, 2018), anthropology (Boyd and Richerson, 1988), psychology (Bronfenbrenner, 1979), economics (Nelson and Winter, 1982), education research (Barron, 2006), and organization theory (Aldrich, 1979; Carroll, 1984; Hannan and Freeman, 1977; Lawrence and Lorsch, 1967; Padgett and Powell, 2012; McPherson, 2004). Although these approaches differ in important ways, they each focus on how populations of individuals adapt to their changing environments and, to a lesser degree, how these populations’ decisions and behaviors shape the environments they inhabit.

Ecological accounts generally treat their phenomenon of interest as an outcome of competition for scarce resources that unfolds in contextualized processes of entity variation and selective retention. The phenomenon of interest may be the morphology of biological species (Darwin, 1859), the proliferation of organizational forms (Hannan and Freeman, 1977), the emergence of stable organizational routines (Nelson and Winter, 1982), the staffing of volunteer organizations (McPherson, 1983), the reproduction of codified rules (Dopfer et al., 2004) and others. In these accounts, the environment selects against individual members of the population depending on traits that are relevant to their survival (Lloyd, 2020). Variation in the traits of individual entities, whether they are organisms, firms, or behavioral patterns, affect how well-adapted those individuals are to their environments: their fitness. Over time, selection eliminates maladapted individuals and their traits, while well-adapted ones are preserved and their traits passed from one generation to the next.

Network ecology, too, treats variation and selective retention as the main mechanism for the coevolution of a population and its environment. In contrast to other ecological accounts, however, network ecology foregrounds entities’ efforts to establish meaningful social relations with others. The scarcity of such resources as time and energy exerts pressures on individuals to be selective in the ties they maintain with others. Over time, maladapted ties and their traits are selected against while well-adapted ones are preserved. The resulting dynamics are modeled with three abstract principles that resonate with classical Darwinist^1^ theory: tie variation, tie selection, and tie retention.

### Elements

The primitive elements of network ecology are straightforward, comprising actors, their settings, and their relations, with each generally matching everyday understandings of the terms. Here, for exactness, we use these terms specifically so as to avoid confusion.

***Settings*** are any socially bounded site for networks and are subject to all of the well-known issues surrounding network boundaries (Laumann et al., 1983). Settings define the range of social actions that are possible in a given situation at a particular time. They establish a time-varying local context in which individuals interact and social relations form and end.

***Social relations*** connect individuals. They encode a normative basis for appropriate modes of interaction. Examples include “lover,” “friend,” “collaborator,” and “rival,” to name a few common interpersonal cases. Relations are institutionalized via relational norms within settings, meaning that the relevant audience of people have expectations about appropriate relation-specific behavior. For example, we are expected to complement lovers, demonstrate loyalty to friends, contribute actively to collaborations, and compete with rivals. Different types of relations constrain each other, either logically, for example enemies cannot simultaneously be friends, or socially: it is difficult to be friends with an ex, for example. Importantly, people sanction violations and reward relational conformity to varying degrees.

***Interactions*** are discrete relational events (Butts, 2008; Lomi and Stadtfeld, 2014). They are the primary observable unit for judging actor conformity to relational norms. Examples include passing a note, holding hands, glaring, talking, joking around, and any other of the thousands of activities that pairs of actors do with each other. They can be casual, such as a fist bump in a hallway at school, or unfold in sequences that are longer, more time intensive and emotionally deep, such as teen lovers talking on the phone, or a friend consoling another over the death of a loved one (Collins, 2004). Actors typically have interactions of varying depth and intensity with multiple peers within a setting. Over time, recurrent, intense, and coordinated patterns of interactions are more likely to become the basis for social relations that inform subsequent interactions (Roethlisberger and Dickson, 2003; Hinde, 1976).

Social relations form when compatible types of interactions either happen with enough repetition (Festinger et al., 1950; Martin, 2009) or with sufficient meaning (Collins, 1993) to build awareness and expectation of further interactions. For example, a fist-bump in a hallway might be a one-off acknowledgement of a good joke; but if it is done every day between students who also joke around and pass notes, then the meaning of the interaction shifts from random sociality to an indicator of friendship. More tellingly, the refusal to participate in an interaction may be perceived as a slight; an outright negation of an underlying relation. Relational outcomes of interactions are fraught with uncertainty and underdetermined; most single interactions are insufficient for determining that a relation exists, and relations must be nurtured to remain active and substantively real. This introduces a degree of local action and variability in social activity beyond relation and networks. It makes stable relations a social accomplishment that requires investments to be maintained and shared history to make meaningful (Collins, 2004).

***Social identities*** encode behavioral profiles that buffer their bearers from ecological selection forces in exchange for adherence to role-specific relational norms. Social identities generalize the meaning of sets of social relations beyond the dyad (White, 2008; Fuhse, 2009, 2021) and invoke generalized expectations about the prospects of interacting among groups (Nadel, 1957; White et al., 1976; Winship and Mandel, 1983). By anchoring expectations about the prospects of successful interactions with (particular types of) others, social identities reduce the information cost of interacting in groups that are too large for everyone to be fully informed about everyone else. More generally, this ability to generalize expectations from a single instance to a set is a precondition for the emergence of complex social systems (Allen and Starr, 2017; Flack, 2017; Luhmann, 1995 [1984]; Simon, 1996).

An encompassing definition of identity exceeds the scope of this paper, as different disciplines associate different meanings with the term. Broadly, the construct we envision establishes a point of exchange between relational sociology (White, 2008) and cognate lines of enquiry into how groups and collectives shape individuals’ behaviors and outcomes (e.g., Akerlof and Kranton, 2010; Tajfel and Turner, 1986; Stets and Burke, 2000). As a starting point, we here consider that social identities are jointly constructed by attributional features and relational configurations. For example, Coleman’s (1961) original notion of a “leading crowd” in schools is a combined set of attributional features, for instance rich student athletes, and relational attributes such as high internal cohesion and strong admiration from others. To the extent that settings admit strong identity divisions, distinct social identities can develop their own relational norms. For example, relational norms about dating might differ between members of a high school leading crowd and an outsider group in the same setting. In institutions and large bureaucracies, we can imagine divisions and occupations creating sub-settings (Lazega et al., 2017; Lazega, 2014), whereas in schools, peer groups are an obvious break with the setting as a whole. More generally, this reflects the well-known idea that nominal network boundaries might not match realist relational boundaries (Laumann et al., 1983; Erikson, 2013). In relational terms, social identity is a fitting analogy to the classic notion of regularly equivalent network position.

Finally, the ***Proximal Environment*** consists of the material, cultural, and institutional features of the environment that shape the settings in which network dynamics occur. The proximal environment appears as fundamentally exogenous to the network dynamics and can be thought of as analogous to a local natural climate. Just as the altitude, rainfall, and average temperature constrain the sorts of plants that grow and the types of relations amongst organisms that thrive in a region, exogenous features shape relational opportunities and activities in the short run. For example, the built environment and natural schedule of each setting limit how often people have the opportunity to interact (Sailer and McCulloh, 2012). Moreover, the long literature on social foci (Feld, 1981) and Blau Space (Blau, 1993; McPherson and Smith, 2019; McPherson, 1983; Brashears, 2008) illustrate the importance of factors that focus attention on individuals who are present or nearby, compatible, and available.

### Dynamics

The dynamics of our ecological theory rests on two basic tenets. The first is to assume that social networks emerge, stabilize, and evolve through stochastic processes of tie variation and selective retention. This assumption offers a rationale for modeling, predicting, and explaining how social networks adapt to changes in their environments. It implies that social ties (i.e., interactions and relations) are exposed to selective ecological pressures, that they vary in fitness over time, and that some ties are therefore robust and stable while others are likely to be selected against. The second assumption is that tie selection operates at hierarchically ordered levels, of which we focus on four: Interactions at the lowest level; relations at the dyadic micro level; social identities at an intermediate meso-level; and the proximal environment at the macro level. Network stability is effectively modeled as a joint function of ecological constraint on relation formation and variation in interactions.

### Tie variation, selection and retention

Successful network ecological models should be able to account for both the emergence of new social systems and the maintenance of well-established ones. The key notion is that settings select for some activities over others and that this process is recursive: past selections shape what is expected of and valuable in future selections. Certainly, deep learning processes are at play in any social system, but it is sufficient for the purposes of explaining system emergence and evolution to conceive of the main activity as selection against stochastic action. At any moment, actors engage in seemingly random activity, some of which is selected for, some other selected against, and the result is a sifted set of social interactions that form a coherent system. Any selective treatment would be sufficient to generate roles and niches as long as the selection features evolve more slowly than the interactions they select for.

Like other ecological theories, our model turns on competition over scarce resources. People have limited time and energy to invest in social relations and seek out basic social benefits. In addition to the dyadic benefits that people acquire through relations, relations are also judged by others: inappropriate relational behavior elicits sanctions that are costly. Tie selection is thus seen mainly as a form of relational discipline: people learn what sorts of activities are acceptable because they are rewarded for acting appropriately and disciplined for acting inappropriately. Neglecting a friend’s joking overture, flirting with a friend’s romantic partner, and being friendly to somebody beneath one’s station will all result in reactions from the alter or the surrounding community that signal the inappropriateness of the behavior. Similarly, helping a friend in need results in gratitude and support that feels rewarding. These micro-level forces of social exchange and interaction condition appropriate behavior (Blau, 1964). Consequently, social contexts encourage some interactions and relations and penalize others.

The stochastic nature of the selection process generates variation both within and between settings in the distribution of relational niches. In culturally well-defined settings such as high schools and government bureaucracies, the general shape of expected relations is well known. But if a relational violation is allowed to stand, it may become normative, shifting the definition of what is acceptable and thus the boundaries of the niche space. For example, stereotypical American high schools place a sharp boundary between “jocks” and “nerds” that rests on translating different skills–athletic brawn vs. intellectual acumen–into relational antagonism. But, if the emphasis in a particular setting is switched such that some athletes show interest in academic achievement and relations that cross this boundary are allowed to stand, then we may see less of the traditional divide than expected from stereotypical understanding. This is what we mean by the emergent accomplishment of relational ecologies: each place will have somewhat unique distributions of niches that reflect their own history of relational disciplinary action. This can occur across actors within a setting, so the divide between football players and computer science students may be stronger than between volleyball players and English enthusiasts. To model the underlying social processes, we differentiate the social contexts in which relational dynamics play out by the temporal rates at which they unfold. This approach, known as timescale separation, is commonly used in the natural sciences to operationalize complex social systems.

### Timescale separation and hierarchical ordering in principle

Our network-ecological framework elaborates the assumption of tie variation, selection, and retention to model the coevolution of social networks with their environment as temporally mediated processes. This is accomplished by defining elements of a network’s social environment in terms of their responsiveness to one another, an approach we adapt from structuralist hierarchy theory (Simon, 1962). Hierarchy theory suggests partitioning the components of complex adaptive phenomena into ordered hierarchies, with lower levels adapting faster to impulses from above than vice versa (Allen and Starr, 2017; Eldredge et al., 2016; Simon, 1996). This implies that what is fixed at one level appears fluid and changing from another; the stability of social processes unfolding in different contexts depends inherently on the timescale taken into consideration. In the natural sciences, this temporal asymmetry is used to limit the complexity of modeling biological, chemical, and physical processes (Flack et al., 2013; Gunawardena, 2014; Simon, 1996). Specifically, timescale separation allows higher-level contexts to be treated as fixed, while lower levels are summarized by equations that describe their steady state (Gunawardena, 2014). Furthermore, timescale separation implies substantive asymmetries in how different levels of context affect each other over time: higher levels constrain lower levels top-down in the short run, whereas lower-level variations affect higher levels bottom-up in the long run. Together, top-down constraint and bottom-up variation characterize how the focal level of network dynamics evolves as part of a broader social system.

Network ecology uses timescale separation to focus on social network dynamics in coevolving social contexts. At the lowest level, we connect to a burgeoning literature that treats fleeting interactions (and relational events) as basis for more durable social relations (Brandes et al., 2009; Kitts et al., 2017; Stadtfeld and Block, 2017; Kitts and Quintane, 2020; Bianchi et al., 2022). An example of an interaction is a passing nod in the hallway; an uncostly and public act of one individual acknowledging another’s presence. More broadly, time spent in the presence of others solidifies the significance of interactions to the point that a relation forms between two individuals that they and others take into account (McFarland et al., 2013). Such relations and combinations thereof present a tested infrastructure around which subsequent fleeting interactions can stabilize into (more or less adaptive) configurations and social networks.

As ties grow more durable, they generalize more readily, and they evolve more slowly, as one moves up from interaction to social relation to group affiliation and the broader setting in which social relations play out. These differences in the rates of variation imply that higher levels summarize information that would be lost as noise in the rapid variation of lower-level interactions (cf., Flack, 2017). Consequently, higher levels orient and constrain lower levels, but not (or less so) vice versa. Time-scale separation thus implies specifiable asymmetries in how different levels of contexts intersect: In the short run, higher levels constrain lower levels: settings constrain identities, identities constrain relations, and relations constrain interactions. In the long run, however, variations that accumulate at lower levels can percolate upwards to mobilize adaptation, provided the conditions are right. Constraints are inherently temporally bounded and subject to erosion from within, just as water flowing across a ridge during a flood can change the course of a river over time; relations that cross seemingly hard boundaries can open a space for new relations of similar sorts to form.

To understand where, when, and how network dynamics shift as tie variation unfolds, it is instructive to quantify how well ties (interactions or relations) among a population (would) align with the setting. To this end, hierarchy theory suggests a distinction between top-down effects of tie selection and bottom-up scope for tie variation.

### Top-down selection: the proximal environment and relational norms

Within a timescale-separated hierarchy of contexts, social network dynamics are constrained top-down by higher levels of contexts that evolve slower than the focal level of relational activities. The highest level at consideration, the proximal environment, can be thought of as analogous to a local natural climate. Just as the altitude, rainfall, and average temperature constrain the sorts of plants that grow and the types of relations amongst organisms that thrive in a region, features exogenous to the network channel access and shape relational opportunities. Such features can be manifold and apply at different levels of generality. For example, people are part of many more or less over-lapping groups, or social circles, that limit which members of those groups come into contact with one another (Simmel, 1908; Blau and Schwartz, 1984; Breiger, 1974). Often, social settings are designed towards the realization of goals other than sustained relational activity, encoding and enforcing communication channels and chains of command that shape interactions and relations among subsets of the population (Bourdieu, 2005; Martin, 2009; Brennecke and Rank, 2016; Rank et al., 2010). More generally, physical or social proximity define the likelihood of two members of a population encountering one another (Feld, 1981; Doehne and Rost, 2021; Adams et al., 2012).

At the level below the proximal environment, enacted relational norms channel relational activities by distinguishing between appropriate and inappropriate social ties. Whereas appropriate ties are well-aligned with the setting, inappropriate ties are at risk of being selected against.^2^ The extent to which ties align with the setting shall be quantified in terms of their fitness. One potentially profitable typology of relational norms turns on alter specificity. *Categorization norms* treat alters as equivalence classes based on attributes and group membership whereas *configurational norms* adjudicate alter’s relations to other ongoing relations. Homophily is the archetypical example of a categorization norm while social balance is the archetype of a configurational norm.

Categorization norms constrain relational activities based on individuals’ salient characteristics and group affiliations. At times, the characteristics of individuals and actual, inferred, or imposed group affiliations come to carry the weight of top-down relational constraint if their meaning is sufficiently well-established and stable within the population (White, 2008; cf. White, 2008 [1965] for an early presentation of networks of categories as ‘catnets’). For example, biological sex is a nearly perfect predictor of non-friendship amongst young children in elementary school, despite them occupying the same physical space. Settings with strong racial antinomy or linguistic differences provide other common examples. When fully routinized, commonly observed patterns in affiliation by category can take on a normative character that transcends specific relations and experiences. If this happens, category membership conditions the appropriateness of particular ties (interactions or relations), effectively constraining lower-level variation in relational activities on the basis of individuals’ assignments to salient categories.

The configurational norms of a setting adjudicate the appropriateness of a particular tie (interaction or relation) based on how the sender and recipient are embedded in the broader network. Social balance is the archetype of a configurational norm, as it asserts that the viability of a tie between two individuals *i* and *j* depends in part on how they relate to others. Specifically, if *i* likes *k* and *j* likes *k,* then *i* will tend to like *j,* as this would balance the triad. However, if *i* likes *k* but *j* dislikes *k* then an unbalanced configuration obtains, diminishing the likelihood of an affective relation between *i* and *j* (Heider, 1958; Johnsen, 1986). More generally, local relational configurations demonstrably make the (de-) activation of some ties more or less likely than would be expected by chance. For example, the insight that friendships in general are characterized by mutuality indicates the salience of a reciprocity norm (Kitts and Leal, 2021; Hruschka, 2010); the fact that friendships tend to cluster locally among individuals who are similar to one another indicates the salience of group norms (Goodreau et al., 2009; Lazarsfeld and Merton, 1954), and that friendship nominations are often directed disproportionately at those with many friends indicates awareness of popularity differentials and status-norms.

For reasons of operability, most statistical network models treat categorical and configurational norms as analytically distinct (as will our illustrative example below), but relational dynamics are arguably convoluted. Often, social categories are associated with role-specific expectations that calibrate their bearers’ scope for acceptable behaviors and relational activities. The role of ‘class clown’, for example, grants its bearer leeway to provoke while maintaining a tenuous connection to the popular clique (Johnson et al., 2003; McFarland, 2004). To further complicate matters, network positions can become reified as social categories, as when popular individuals with many incoming friendships come to be known as ‘the in-crowd’, or when isolated individuals are stigmatized as ‘outsiders’ or ‘loners’. If such labels become attached to individuals, they may limit those individuals’ scope for relational activities, thereby channeling tie variation in a setting. To acknowledge ensuant social complexities, we refer to interconnected categorical and configurational norms as ‘social identities’ and conceive of the higher-level social context in which relational dynamics unfold as a system of identities competing for representation in society more broadly (White, 2008), i.e., across settings and over long timeframes. A product of their culture and their time rather than of any one particular setting, social identities encode generalized expectations about role-appropriate behaviors and relational activities given an individual’s assigned, claimed, or imposed identity.

By acknowledging the possibility of multiple salient social identities in a setting, network ecology introduces a demographic component to social network analysis. To understand and predict relational dynamics, it is necessary to consider the generalized expectations that are attached to different (species of) social identities. That is, just as natural ecologists are rarely interested in the life-story of a particular bear or fish and concerned instead with “top predator” and “keystone species,” we ultimately seek to understand how social identities shape the relational norms of a setting and the relational outcomes that obtain. At this point, we leave a further elaboration of concepts of social identity to future work and consider instead the lower-level resource base in which identities can form and relational dynamics unfold: relational niches and the activation potential of dyads.

### Bottom-up variation: relational niches and the activation potential of dyads

While top-down selective constraint captures important aspects of relational dynamics in social networks, it does not fully determine the outcomes that obtain. Structural complexities and ambivalences render the reproduction of stable social networks uncertain, as actors navigate and shape the configurations they are a part of (Sewell, 1992). In nontrivial ways, social settings coevolve with the networks that occupy them and with the lower-level activities of social actors pursuing their respective, idiosyncratic goals. In the long run, relational norms and group identity are subject to changes, be this because of compositional changes in the groups (Breiger, 1974) or because of ongoing lower-level efforts to shape the setting to one’s advantage (Padgett and Ansell, 1993). After all, social actors not only attend events but organize them together (Block, 2018), they not only join conversations but also leave them (Hoffman et al., 2020) or change topics, and they not only work in institutions, but they also shape those institutions to their purposes (Lazega et al., 2017). Consequently, social networks are at least partially malleable; within limits, they enable social actors in a population to exploit and adapt the given setting to their respective purposes.

At the network level, the normative constraint that the network of enacted relations impose on the members of a population leaves room for interpretation and some degree of subculture construction in interactions (e.g., Fine, 1979; Hebdige, 1979), which is evidenced as tie variation in interactions. In interactions persons learn to adapt and enter new modes of relating and this can percolate out and have moderating effects on both the networks and the contexts. For example, a preadolescent relational norm not to befriend members of the other sex constrains relational preferences even while children of either sex do interact on occasion; working on school assignments and during breaks creates opportunities for encounters to intensify into social relations over time. Moreover, macrostructures entail multiple types of ties and pressures, often at cross-purposes (Sewell, 1992). The resultant relational uncertainty leaves room for lower-level tie variation: multiple possible local configurations that are similarly fit, need not resemble each other, and can proliferate and diffuse within and across settings over time.

At each level of analysis, timescale separation implies that higher-order constraints will vary in their selective strength, with some features being nearly absolute and others admitting leakage around the edges. Such differential selective pressures build within settings over time, which helps account for why we would generally expect the emergence of different macro-structures from similar relational micro-mechanisms. To operationalize this idea, we introduce the concept of tie fitness as a metric that quantifies how well any particular tie (interaction or relation) among members of the population (would) align with the relational norms that are expressed in the setting.

### Tie fitness as a time-varying metric of relational alignment with a setting

The core concept for modeling ecological constraint and residual scope for tie variation is relational fitness. Relational fitness is a joint accomplishment of the pattern of other relations at the surface level and the needs that the relation in question fulfills at a deep level. On the surface, relational fitness refers to the extent to which a tie (interaction or relation) aligns with the expressed relational norms of the setting. By this first definition, a relation fits if it meets the appropriateness standards that are reflected in the aggregate of observed relations of that type. This is our operational definition below and hinges on the boot-strapped nature of relations within a setting. Fit ties prove themselves in repeated interactions and thus stabilize into durable social relations. To understand why settings differ in their emphases on relational norms, this definition will suffice, though it does not tell us why expectations for one set of relations form over another other than via historical inertia.

At a deeper level, tie fitness is shaped by the benefits that realized relations provide to the actors involved. Specifying such deeper needs requires a theory of social agency that considers actors’ context-dependent motivations alongside patterns of enacted behaviors (Lazega and Pattison, 1999). For example, it is not simply appropriate that a bear eats salmon, though finding that some sorts of animals routinely eat other types of animals is a convenient way of understanding the surface-level dynamics of complex ecological systems (Poisot et al., 2012; Thébault and Fontaine, 2010; Borrett et al., 2006). At a deeper level, salmon provides a concentrated source of calories for bears that, in turn, provides them with the energy needed to make and raise more bears. Any other convenient source of concentrated calories, particularly ones less risky to obtain, would do just as well. By analogy, relations must provide resources that are fundamental to the social being of each actor, viz. their identity: teens need friends for emotional support and a sense of belonging, non-profit organizations need volunteers willing to work for them, and leaders need followers to do their bidding. This deeper understanding of fitness is important for conceptualizing the normative bases of tie selection and how the relational system evolves. Just as a campground garbage pail is a simpler source of calories for bears than swift moving fish, new interaction forms will shift the ways that people engage with each other. The rapid rise of social media, for example, has changed the ways that teens engage with each other but has probably not changed the fundamental needs that these interactions are filling. But, just like garbage in the woods for the life of bears, such changes might not be in the long-term interest of the actor.

Unlike calories in natural ecosystems, the benefits of social relations depend on the setting: the set of benefits for adolescents in schools will be very different from that for partners in a law firm. But within the conditions of any given setting, actors are willing to make the effort necessary to maintain relations because these provide benefits that are only obtainable through repeated interactions. One promise of network ecology going forward is to probe below the surface of the relational system to characterize the meanings and rewards that relations provide. In so doing, we hope to characterize whole network ecologies by the corresponding flows of resources through the system, whether these are social support in the case of friendship, valuable advice in mentoring, or profitable exchange in market transactions. As illustrative case, we next consider effects of tie fitness on adolescent friendship.

## The fitness of adolescent friendship as illustrative case

### The relational system of adolescent friendship

The discussion above has outlined general features of an ecological account of networks and is intended to sketch basic elements and mechanics of the framework. We next develop these points for adolescent friendship as an illustrative case. The substantive setting in which adolescent friendships play out is generally schools, though occasionally the empirical data limit is within cohorts, grades, or classrooms. Schools are rich socially because they are sequestered and segregated from much of the adult world (Coleman, 1961; Waller, 1965 [1932]). Friendship networks in schools have been widely studied and thus there is a good general sense of top-down constraint, as well as of the selection forces and of the implicit relational norms that shape interactions in the relational system.

We focus here on self-reported friendship relations of the sort collected when researchers ask students to name friends on a sociometric survey. These are nominal friendships, though they may not be phenomenologically friendships. Irrespective of their true status, analyses of networks of self-reported friendships routinely uncover recurrent relational configurations; notably that friendships tend to be reciprocated (Kitts and Leal, 2021; Hruschka, 2010; Hartup and Stevens, 1997), that friendships cluster locally in groups that align on salient characteristics (McPherson and Smith-Lovin, 2001; Goodreau et al., 2009; Lazarsfeld and Merton, 1954; Wimmer and Lewis, 2010), and that nominations are disproportionately directed to some individuals, resulting in popularity- and status-inequality (Smith and Faris, 2015; Faris and Felmlee, 2011). These configurational norms, together with three potential markers of social identity (self-reported sex,^3^ ethnicity, and socioeconomic status), form the basis for our model of the relational system and allow us to infer a surface-level fitness metric.

As we developed in the first part of this paper, tie fitness quantifies how well a particular tie (interaction or relation) aligns with higher-level selection pressures (relational norms) that set the ‘tone’ of the setting. For example, a centralized setting in which a few individuals attract many incoming nominations assigns high fitness to ties that connect to popular individuals and low fitness to ties that are directed to outsiders. In contrast, locally clustered settings encourage transitive closure and select against ties that are not embedded in groups. These overarching selection pressures can be summarized by a fitness function comprising the effects of relational norms and social identities as inferred from the set of all reported relations. This surface-level definition of tie fitness is sufficient for operationalization if we assume that settings are mostly stable and that patterns of past relations are thus good indicators of patterns of new relations. To illustrate how fitness affects relational dynamics, we draw on data that has been collected as part of a large longitudinal study of adolescent social networks.

### Dataset

We use data obtained from the PROSPER study (Spoth et al., 2011), which tracked two cohorts of students in 28 schools in Iowa and Pennsylvania from grades six through twelve. Across eight measurements (T1–T8; two measurements were done in grade six), study participants nominated friends from full rosters of fellow cohort members. Thirteen schools where students attended separate elementary schools were excluded from our analysis, as this factor complicates analyses without contributing to our illustrative objectives. We excluded one setting with fewer than eight measurements. Consequently, the final database for the following analyses consists of 8 measurements each of 2 cohorts in 18 schools, i.e., 288 adolescent friendship networks collected in 36 distinct settings. Several of the following analyses predict the future state of reported relations (e.g., new tie formation, tie dissolution, and tie longevity). Since it is impossible to determine the future status of relations reported at the last measurement, T8, we limit our analyses to the 252 networks at T1 to T7.

### Method

The following analyses proceed in two steps. First, we identify patterns in reported relations that enable the inference of a surface-level fitness function. This fitness function quantifies how well a relation among any two members of the population would align with the norms that are jointly expressed in the set of all reported relations. Fit ties conform to and reproduce the relational norms of the setting, whereas unfit ties are misaligned with the setting and at risk of being selected against.^4^ For example, in a setting that turns on reciprocity, a reported tie *e*_*ji*_ should, ceteris paribus, increase the fitness of tie *e*_*ij*_. Similarly, in a setting that encourages densely interconnected peer groups, having shared mutual acquaintances should, ceteris paribus, increase the fitness of ties *e*_*ij*_ and *e*_*ji*_. We operationalize this surface-level metric of tie fitness as the predicted probability of observing a particular relation.

Numerous statistical network models are available to predict the probability of two nodes in a network being connected given a set of reported relations (for many, e.g., Lusher et al., 2012; Robins et al., 2007), the relational goals of actors (Snijders et al., 2010), and/or the sequences in which relational dynamics play out (Butts, 2008; Stadtfeld et al., 2017); potentially at different levels of contexts (Lomi et al., 2016; Lazega and Snijders, 2016; Wang et al., 2013; Snijders, 2016). The choice among frameworks ultimately involves tradeoffs between precision of estimation, underlying assumptions, computational intensity, and flexibility. As our primary interest is in specifying model coefficients by which to predict tie probabilities, we ultimately favored a network logit model for its relative simplicity, scalability, and flexibility (Almquist and Butts, 2014; Block et al., 2018). We acknowledge that this model underestimates standard errors but since we are unconcerned with estimating significance levels, this drawback does not affect our aims. Furthermore, this framework enables us to simulate scenarios in which subsets of the population prioritize relational objectives that contravene the relational norms of the setting (see Observation 5 in the next section).

Specifically, to quantify the probability of a tie being reported between any two nodes, we fit generalized logistic regression models to the network data. These models estimate the likelihood that individual *i* reports a social relation with individual *j* at measurement *t* given relational norms that are inferred from the graph of reported relations among all members of the population, *G*_*t*_(*V, E*), with random effects included to control for latent characteristics of the senders and recipients of relations:

(1) $$\text{log}\left(\frac{p\left(e_{ij}|G_{t}\right)}{1-p\left(e_{ij}|G_{t}\right)}\right)=\sum \left(\beta_{C}\cdot x_{ij}\right)+\sum \left(\beta_{R}\cdot x_{ij}\right)+\varepsilon_{S}+\varepsilon_{R}$$

where *β*_*C*_ is a coefficient vector summarizing the effects of categorization norms and *β*_*R*_ is a coefficient vector summarizing the effects of relational norms. As categorization norms we consider homophily on self-reported sex (male/female), on ethnicity (distinguishing between majority and minority group), and on parental socio-economic status. The configurational norms turn on three dimensions: status-orientation as operationalized by indegree popularity (*β*_1_), local clustering as operationalized by the number of shared mutual acquaintances (*β*_2_), and reciprocity(*β*_3_). At each measurement *t*, the corresponding covariate matrices obtain by appropriate transformations of the network graph’s adjacency matrix, *R*_*t*_:

$$x_{1,ij}=\text{log}\left(C_{j,t}+1\right);x_{2,ij}=\text{log}\left({\left|R_{t}\right|}^{2}+1\right);x_{3,ij}=R_{t}^{T}$$

with $C_{ij}=\sum_{j}x_{k\neq i}$ the column-sum of R denoting *j*’s indegree popularity (correcting for incoming relations from ego, *i*), *x*_2*,t*_ quantifying local clustering by the log of (one plus) the number of two-paths connecting *i*→*j* in the symmetrized adjacency matrix, $\left|R_{t}\right|=R_{t}\cup R_{t}^{T}$, and reciprocity identified by the transpose of the adjacency matrix of relations, *R*_*t*_. To facilitate comparisons between measurements, we standardized indegree popularity and local clustering for each measurement (z-scores). Table 1 reports coefficient estimates for one setting, CM116C2, for measurements T1 through T7.

Consistent with the other networks in our study and with many other studies of adolescent friendship networks, the friendship system in setting CM116C2 revolved around consistently strong, positive relational norms for clustering, reciprocity, and status-orientation. Although not the primary aim of this paper, we do briefly consider patterns in model coefficients across the 252 networks in our sample in the following section. Before we do, however, we must first address how the generated output is used to quantify dyadic fitness. Having fitted regressions to each of the **252** networks, the probability of relations being reported among any of the possible dyads in each network obtain by reentering the data into Eq. (1) and converting the resulting logits into predicted probabilities, i.e., retrieving *p*(*e*_*ij*_|*G*_*t*_) for any two individuals *i* and *j* in the population. Conceptually, these predicted probabilities quantify how well a tie *e*_*ij*_ would align with the relational norms of the setting. As absolute probabilities depend on population size, we rank-normalized them for each network so that the dyadic tie with the highest probability in each network is assigned a fitness of *f* = 1, the dyad with the lowest probability is assigned *f* = 0, and all other ties are arrayed in between. A tie’s fitness *f* thus amounts to its percentile rank (PR) in predicted probability within the population at that measure: *f* (*e*_*ij*_|*G*_*t*_) = *PR*(*p*(*e*_*ij*_|*G*_*t*_)). Thereby, each dyadic pairing within the population is assigned a fitness score *f* that ranges from 0 to 1 and amounts to its fitness percentile rank among all potential ties in the population.

**Fig. 1.**
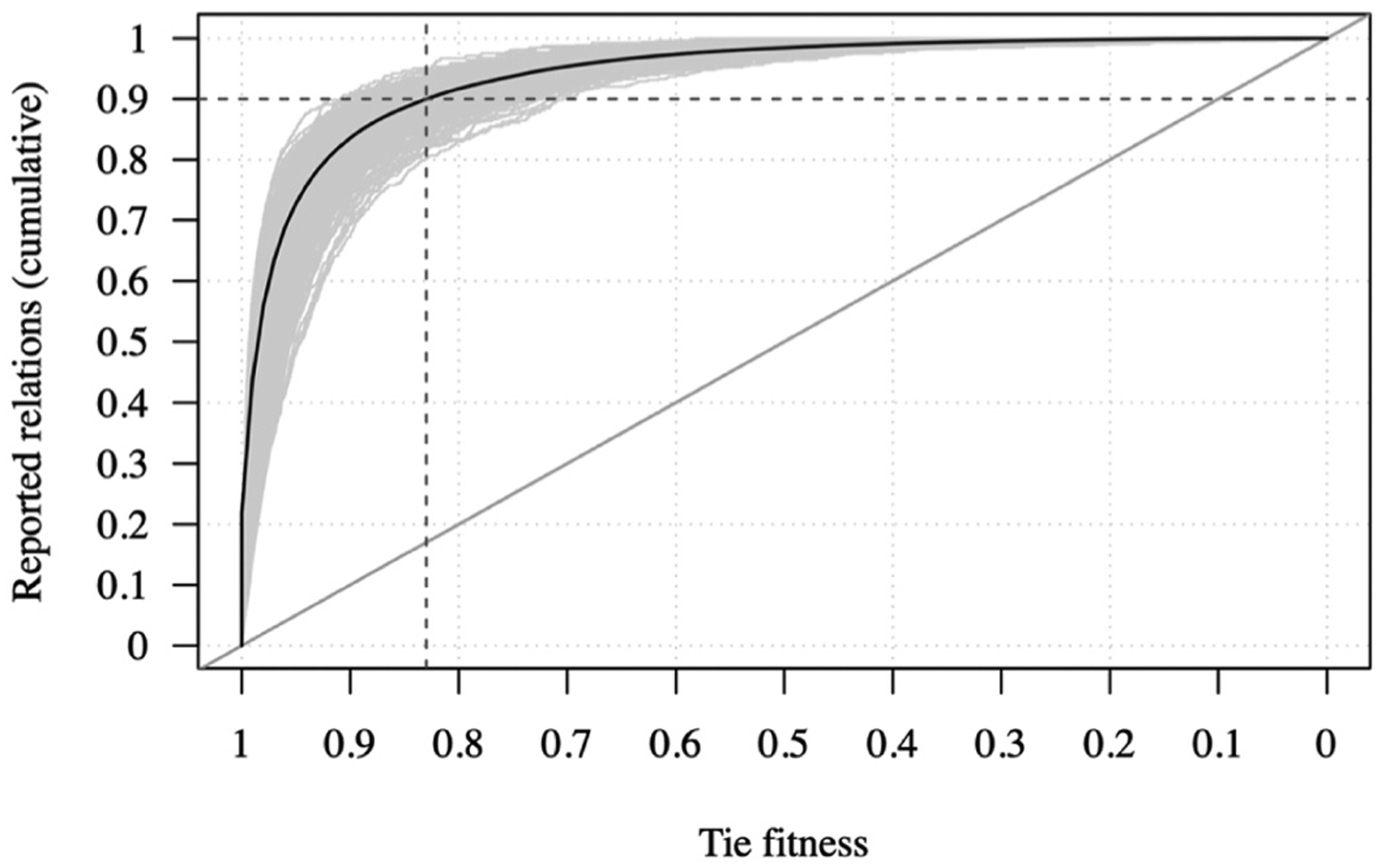
Cumulative share of reported relations by tie fitness.

As we developed above, the network-ecological framework builds on the premise that poorly adapted ties are exposed to ecological selection pressures that limit their ongoing viability and longevity. For example, an expressed friendship that is considered inappropriate to the setting may result in ridicule and peer pressure to end the relation. However, higher-level ecological forces fail to account for lower-level dyadic idiosyncrasies such as a shared history or personal sympathy. This suggests some scope for lower-level variability, which we operationalize by adjusting a threshold at which ties transition from being well-aligned to being misaligned with the setting. To establish this threshold, we consider the fitness of reported relations. For established relational systems such as adolescent friendships in schools, we expect most reported relations to be well-adapted and only a few reported relations to be misaligned. For example, assuming 10% of reported friendship relations are misaligned across settings on average, we can specify as ‘fitness threshold’ the 90th percentile of the fitness of all reported ties in our sample. To evaluate this threshold, Fig. 1 depicts the cumulative share of reported relations by fitness for all 255 networks.

The y-axis in Fig. 1 denotes the share of reported relations in each network that have a fitness score equal to or greater than the corresponding point on the x-axis. Each of the 252 networks is depicted as a separate line. Moving from left to right on the graph, a steep increase in the line indicates a strong correlation between that fitness level and the likelihood of a relation being reported. The solid dark curve tracks the cumulative share of reported relations by fitness averaged across all networks. It shows that 90.3% of reported relations across all measurements are assigned fitness scores of *f* ≥ .83 (cf. the intersection of dashed lines in Fig. 1). As this threshold, the worst-performing model identifies 80% of reported relations as fit; the best-performing model identifies 97%. This high level of retrieval is hardly surprising, as the models were primed on the reported relations. What is interesting, however, is to consider (a) whether factors that influence tie fitness are consistent across networks, thereby indicating a basis for a general ecological account of adolescent friendship, and (b) whether and how fitness informs relational dynamics, notably new tie formation, stability, and longevity. To this end, we relate tie fitness back into each network.

**Fig. 2.**
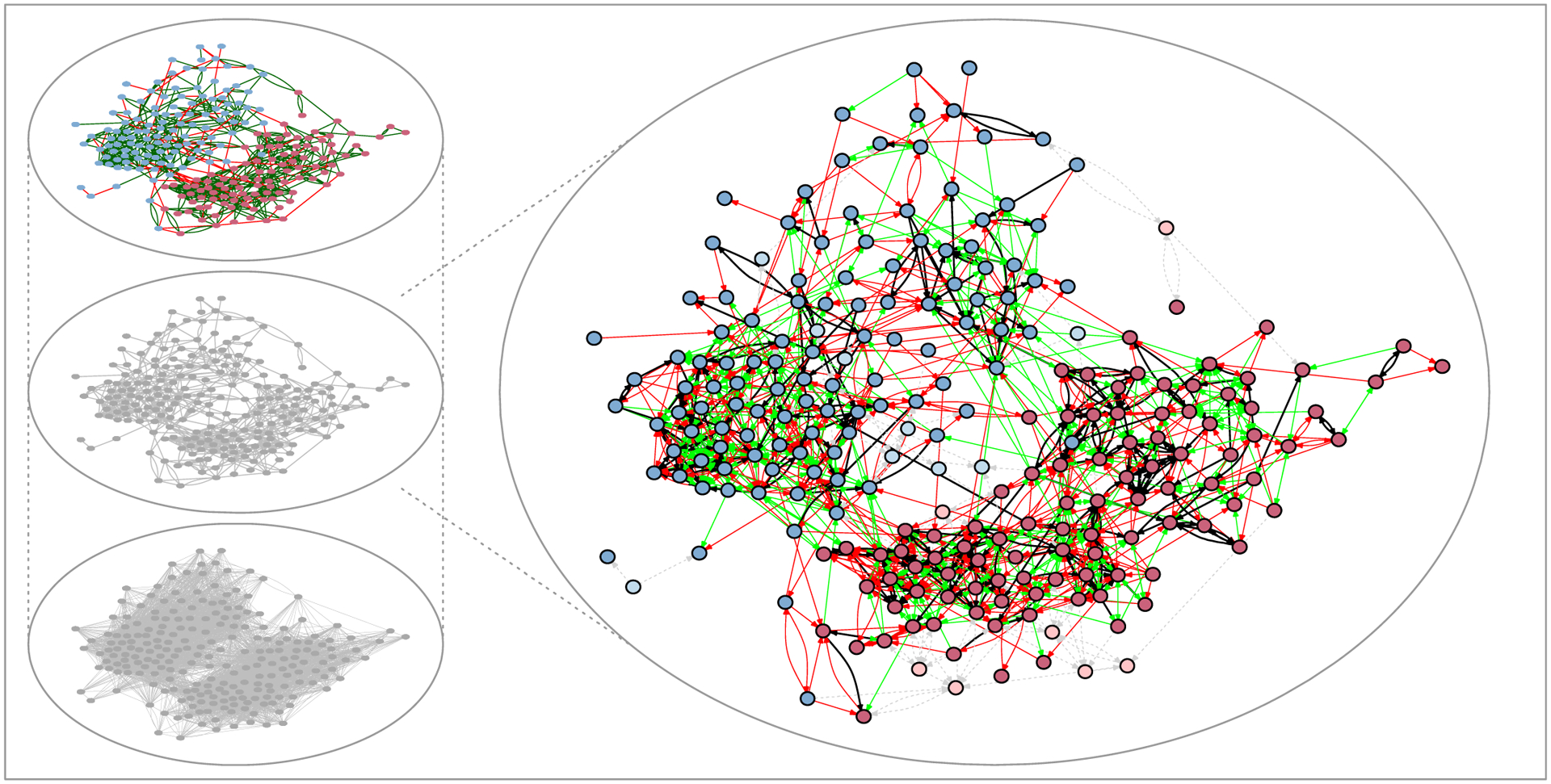
Social Network with surface-level fitness metric encoded, example. Note: The stacked networks on the left show the timescale-separated structure of setting CM116C2 at measurement T3. The middle inset depicts the network of social relations reported in T3. The bottom inset shows all dyads that would be fit ties if activated, representing the potential for interactions that are well-aligned with the relational system. The top inset depicts current and future relations (at T4), with red edges identifying unfit ties that are subject to selection. Males are colorcoded in blue, females in mauve. In the large ellipse to the right, thick black edges identify relations that will be reported again at T4, red edges identify relations that will not be reported at T4, and light green edges identify dyads that will first be reported at T4. Light coloring of nodes identifies individuals who did not participate at T4. As those individuals’ outgoing ties’ future status are unknown, they are presented as dashed gray edges (and excluded from analyses).

Fig. 2 presents a network with fitness information encoded for different levels. Insets on the left of the figure display three levels of social complexity that play out at different timescales: The central inset depicts the reported network at the time of measurement at a meso-level, wedged between the higher-level relational norms and constraints that shape relational dynamics (from above) and the opportunities for lower-level variation in interactions (below). The bottom inset depicts all fit dyads in the setting (*f* ≥ .83). If activated in interactions or social relations, these ties would align with the setting’s relational norms and would not be selected against. In effect, the bottom inset depicts the scope for lower-level tie variability (in interactions or expressed social relations), i.e., the activation potential of the setting. The top inset visualizes the expressed relational norms of the setting in terms of the fitness status of each reported relation: fit relations are color-coded in green, unfit relations in red. The enlarged network, on the right, shows the status of reported relations using different colors: ties that will be reported again at the next measurement are dark green, ties that will not be reported again at the next measurement are colored in red, and dyads that will first be reported at the next measurement are added in light green. Dashed gray ties denote reported relations whose future status is unknown because the reporting individual did not participate in the study at the following measurement. Understanding how fitness affects these various relational outcomes is a fundamental step in comprehending network dynamics through an ecological lens.

To unpack Fig. 2, the following section develops five observations about network dynamics that are suggested by the proposed network-ecological framework. Following a discussion of the salient categorization- and configurational norms that characterize the 36 settings over time (Observations 1 and 2), we elaborate on how tie fitness predicts new tie formation, stability, and longevity (Observation 3). We then introduce the relational niche as a general mapping of the scope for tie variation that individuals can exploit to pursue their idiosyncratic relational projects (Observation 4). Finally, a relational niche’s capacity to accommodate individuals with divergent relational goals introduces demographic diversity in social identities and relational norm heterogeneity as context-dependent drivers of network dynamics (Observation 5). Together, these observations suggest an ecological explanation for the macro-variation puzzle that we posed in the introduction.

### Five observations on the network ecology of adolescent friendship

#### Observation 1: The time-varying salience of categorization friendship norms

The longitudinal data allow us to consider how categorization norms evolve over time. On a surface level, categorization norms manifest themselves in mixing patterns among and between categories in ways that depart systematically from mixing patterns that would be expected by chance. To keep matters simple we here focus on nominal characteristics; future work may consider the time-varying salience of reified network positions as social identities (such as protracted (non-)mixing among and between the ‘in-crowd’ and ‘outsiders’, etc.). Nominal assortativity is a commonly used measure of the degree to which individuals with similar characteristics tend to interact (Newman, 2003). An assortativity coefficient of 0 indicates that interactions are randomly distributed in line with the overall demographics of the population while a coefficient of 1 indicates only within-group interactions. Table 2 reports average and extreme assortativity coefficients across settings and over time, for assortativity on parental socioeconomic status, ethnicity, and self-reported sex.

Across settings, the data suggest only moderate levels of assortativity on parental socioeconomic status (SES) and some assortativity on ethnicity. Of 252 analyzed networks, only six exhibited assortativity levels greater than.3 on parental SES, while 16 showed assortativity levels greater than.3 on ethnicity. Among the latter, 10 are accounted for by two outlier settings (CM111C2 and CM224C2). In contrast to ethnicity and parental SES, the networks consistently showed substantial assortativity by self-reported sex across measurements. In Grade 6 (measurements T1, T2), relations were particularly sex-segregated, with boys nominating boys and girls nominating girls as their friends across all 36 settings. However, the strength of sex-based homophily decreased markedly over time; from 92.2% in Grades 6/7–86.4% by Grades 10/11 (N = 95,959). A concurrent decline in sex-based assortativity was observed in 35 of the 36 settings (cf. Table 1).

We note that the moderate levels of assortativity on parental SES and ethnicity may well be due to limitations in the operationalization of these characteristics. In contrast, the systematic decline in sex-based assortativity across settings does suggest a set of (well-established) ecological mechanisms that apply across school settings, such as the onset of puberty, the transition from elementary- to middle- to high-school, and the age-specificity of North American cultural norms about the appropriateness of cross-sex friendships. Together, these features of the proximal environment shape the developmental trajectory of adolescent friendships at the cohort level. Without further elaborating, we point to the importance of peer groups, cultural proscriptions, and individuals’ physiological changes in shaping these relational dynamics (Telzer et al., 2015; Connolly et al., 1999; Lempers and Clark-Lempers, 1993). Going forward, one task of network ecology is to consider how such forces interact with broader institutional and cultural environments to shape categorization norms within and between settings.

#### Observation 2: Configurational friendship norms are broadly consistent across settings

**Fig. 3.**
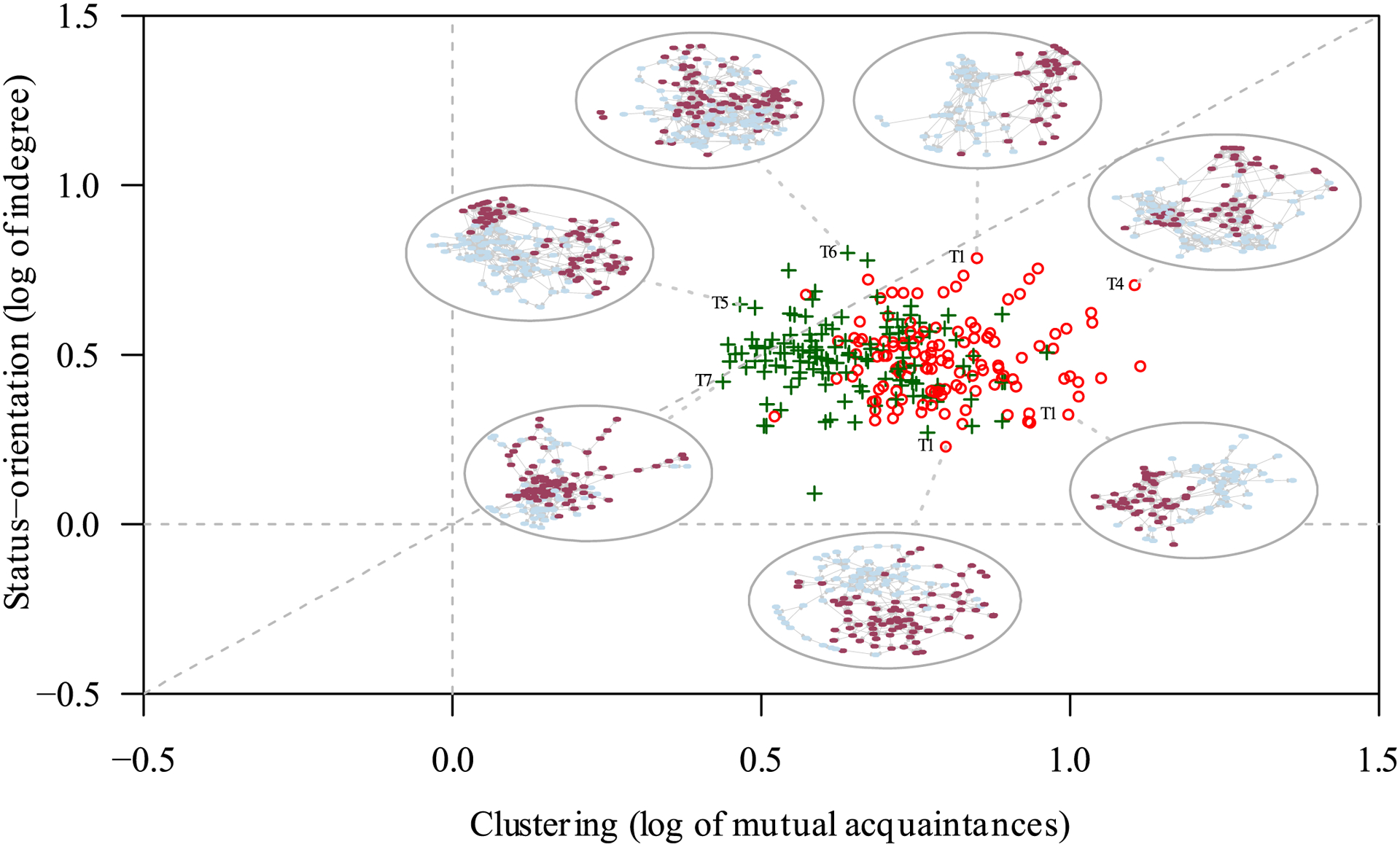
Coefficient estimates for clustering and status-orientation. Note: Each point on the scatterplot represents the coefficient estimates for status-orientation and clustering for one network measurement (N = 252). Networks with an above-median reciprocity coefficient are represented by green plus signs, networks with a below-median reciprocity coefficient are represented by red circles. The top left inset (a) tracks the share of reciprocated relations across measurments, with gray shading identifying the lower and upper decile. All coefficients are significant at p < .001.

While the salience of sex as a marker of social identity changes reliably with study participants’ age, the modeled configurational norms remain broadly consistent over time. Across all 36 settings and measurements, reported friendships tend to be reciprocated, they tend to cluster locally, and they tend to go to those who are nominated by many others (status-orientation). In line with prior research, all three coefficients are substantial and positive for all 252 network measurements. Fig. 3 plots coefficient estimates for local clustering (log of mutual partners, z-transformed) on the x-axis and for status-orientation (log of indegree, z-transformed) on the y-axis for each network measurement.

For our purposes, Fig. 3 conveys three main insights. First, it shows that the 255 network measurements each yield similar coefficient estimates for clustering and in-degree. At 19.2% and 22.6% respectively, the coefficients of variation indicate that the estimates for either model parameter fall closely around their respective means of 0.72 and 0.49. Second, the inset network visualizations showcase that even modest differences in global coefficient estimates can reflect substantial variation in macro-topology. Third, the observation that networks with high levels of reciprocity have low clustering acknowledges the well-known methodological challenge that nested network model terms can result in unstable estimates. This is because higher-order configurations such as transitive closure may be partly explainable by lower-order configurations such as mutuality (e.g., Faust, 2010). For example, a configuration in which individuals *i*, *j*, and *k* each list the others as friends is both reciprocal and transitive, i.e., each dyad has their relation reciprocated and each has the third as mutual acquaintance. It is therefore initially unclear whether a reciprocated friendship that is embedded in a dense cluster of relations indicates the presence of a reciprocity norm, of a clustering norm, or of a different norm entirely. Indeed, in our illustrative case, measurements with high reciprocity coefficients have lower coefficients on clustering, placing them to the left of the scatterplot presented in Fig. 3 (settings with above-median reciprocity coefficients are identified by green ‘ + ‘ signs).

As a challenge to statistical analyses, interdependencies among relational mechanisms are well known from the literature on random graphs and ERGM degeneracy (Handcock et al., 2003; Hunter et al., 2008; Snijders et al., 2006). Although several methods are being developed to resolve these issues from a statistical point of view, network ecology also points to a substantive interpretation: a nestedness of lower-level configurational norms in higher-level norms suggests the possibility of nonlinear accumulative effects when, say, a clustering norm is amplified in a setting that also emphasizes reciprocity, or vice versa. In such cases, small differences in relative emphasis can result in widely different macro-topologies (McFarland et al., 2014). Furthermore, timescale separation suggests that indeterminacies associated with the true drivers of relational dynamics create opportunities for lower-level variability in relational outcomes, enabling social actors to shape local configurations to their purposes and interests.

To develop this idea further, the remaining three observations shift attention from comparisons of model coefficients to the scope for variation in relational outcomes that can be predicted for a population within a given setting. Specifically, we use the statistical models discussed above to quantify the (surface-level) fitness of current or potential social ties between any two members of each sampled population. As a proof-of-mechanism test, we use this dyadic fitness metric to predict the formation, survival, and longevity of reported relations (Observation 3). Then, we identify clusters of ‘fit’ ties in the population. These clusters bundle relational activity within the population and can therefore be seen as fecund relational niches (Observation 4). Finally, we simulate lower-level tie formation dynamics to quantify the scope for norm-divergent relational activity that relational niches allow for, effectively introducing diversity in social identities and relational norm heterogeneity into the analysis of social networks (Observation 5). Together, these observations suggest a second, demographic approach to addressing the macro-variation puzzle that was posed in the introduction.

#### Observation 3: Fitness predicts new tie formation, stability, and longevity

We use the statistical models discussed above to quantify the predicted probability of a tie being reported among each possible dyad in the population. As outlined above, we take the rank-ordering of probabilities as a surface-level definition of fitness that expresses how well each dyadic tie (would) align with the relational norms of the setting. Repeated measurements in stable populations allow us to test the efficacy of this fitness metric as a predictor of new tie formation, stability (survival), and longevity. To this end, we fit a set of generalized linear models to predict the future status of relations as functions of dyadic fitness. The output of these models is presented in Table 3.

**Fig. 4.**
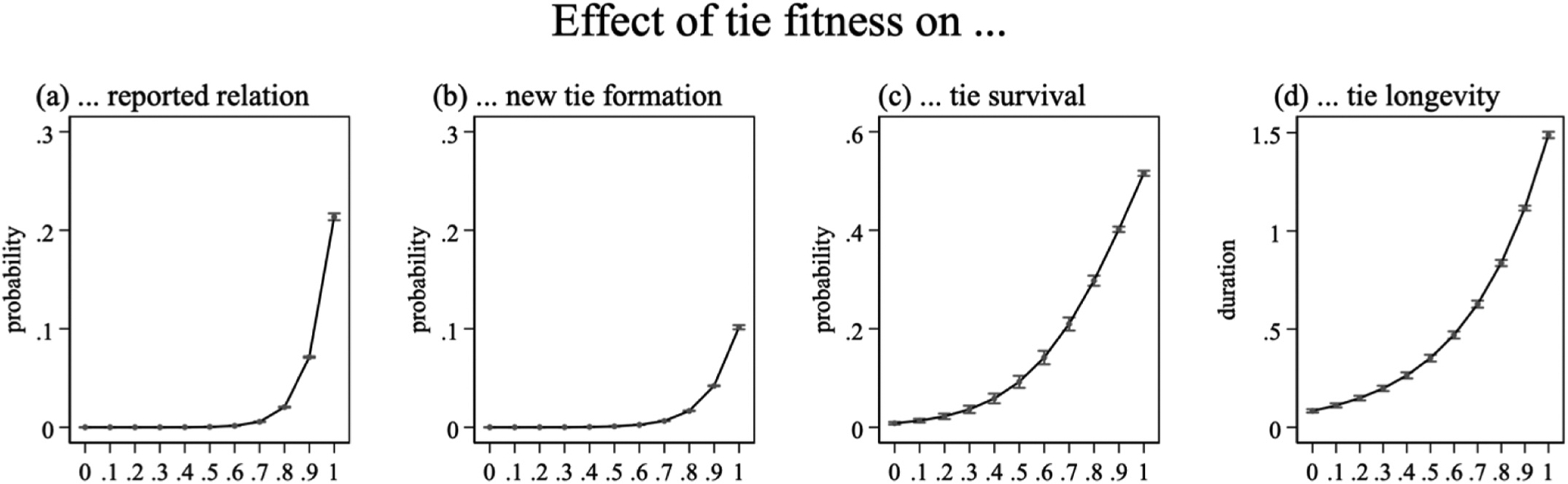
Fitness predicts reporting, formation, survival, and longevity of adolescent friendships. Note: Marginal effects of tie fitness on outcomes derived from the Models presented in Table 3. Tie fitness is plot on the x-axis.

Across measurements, we find positive effects of fitness on the likelihood of a tie being reported as relation (Model M3a), its newly being reported as a relation at the following measurement (Model M3b), a reported relation’s prospects of survival (Model M3c) and the longevity of reported relations (Model M3d). Fig. 4 visualizes the marginal effect of fitness on these aspects of social relationships.

As expected, ‘fit’ ties are more likely to be reported as relations: around 25% of ties in the 100th fitness percentile are predicted to be reported as relations (Panel a). This is supported by the data, as 21.9% of all relations in the 95th fitness percentile are reported (N = 460,226). As fitness derives from the maximum likelihood of observing reported relations, it would be surprising if this were otherwise (there is, in short, a built-in dependency between tie fitness as it is inferred from reported relations and the dependent variable of M3a: whether or not a tie is reported). More tellingly, fitness also strongly increases the likelihood that a currently unreported dyad will be reported as a friendship at the next measurement (Panel b). Specifically, Model M3b predicts that 10% of all unreported dyads in the 100th fitness percentile will be named as friends in the next period. Again, this is supported by the data: 10.7% of unexpressed dyads with fitness f> .95 will be reported in the following period (38,378 of N = 359,311). Conversely, only 0.23% of unfit dyads (f<.83) will be newly reported in the following period (10,131 of N = 4014,122). Despite substantial turnover in the relations that are nominated from one period to the next, the overall relational system turns on a subset of ‘fit’ ties; the vast majority of unfit dyads never being reported as friends.

Fitness also predicts the longevity (Panel c) and survival prospects of reported relations (Panel d), even when accounting for friendship quality. Specifically, Panel (c) predicts that ties in the 100th fitness percentile will be reported 1.49 times in the future. By comparison, relations of median fitness are predicted to be reported 0.23 times. In line with this finding, only 12.1% of all relations with below-median fitness in our sample are reported again in the next period (N = 1697). Contrast this with 53.1% of all reported relations in the 95th fitness percentile (N = 82,490). To a degree, this divergence between the longevity of fit vs. unfit ties seems attributable to the quality of the reported relation: 81.5% of relations that survey respondents reported as their ‘best friends’ are very fit (>.95), compared with 67.8% of regular friends. On average, best friends will be reported 1.71 times in the future, and 62.3% of best friends are reported from one period to the next (N = 39,337).

Overall, we consider the finding that fitness predicts new tie formation, self-reported tie quality, survival, and longevity as a proof-of-mechanism test aimed mainly at building intuition: the presented surface-level definition of tie fitness broadly predicts the social dynamics that we associate with friendship relations. Moving forward, we note that most of the reported relations score highly on fitness but that not all fit ties are reported as relations. These fit but dormant ties introduce a quantifiable scope for tie variation, or activation potential, within which viable relational dynamics play out. To elaborate this point, we consider how that activation potential is distributed within the population. To this end, Observation 4 introduces the concept of ‘relational niche’ as a broad analogy to the ecological niche.

#### Observation 4: Relational niches and the activation potential of dormant ties

Observation 3 has established connections between tie fitness and relational outcomes in the investigated settings. However, despite most *reported ties* being fit (primarily due to how the surface-level metric has been constructed!), most *fit dyads* are unreported and will remain so in the future. As illustration, consider a population of 100 students, each reporting four friendships on average. If 90% of reported ties are fit at the level of f≥ .83(as seen in our sample of networks, cf. Fig. 1), this amounts to 368 out of the 400 reported relations. However, these 368 reported relations only represent 21.9% of all 1, 683 fit dyads in the population (17% of 9900). Consequently, four out of every five fit dyads are not reported as relations, even though they would be well-aligned with the setting. We refer to unreported fit dyads as ‘dormant ties’ to signify their status as untapped resource base for viable relational activity. Just as mutual acquaintances can establish ‘weak’ ties that are socially meaningful even in the absence of an expressed relation (Granovetter, 1973), a dormant tie engrains an activation potential that would, *if expressed*, support tie experimentation, variation, and ensuant relational dynamics.

**Fig. 5.**
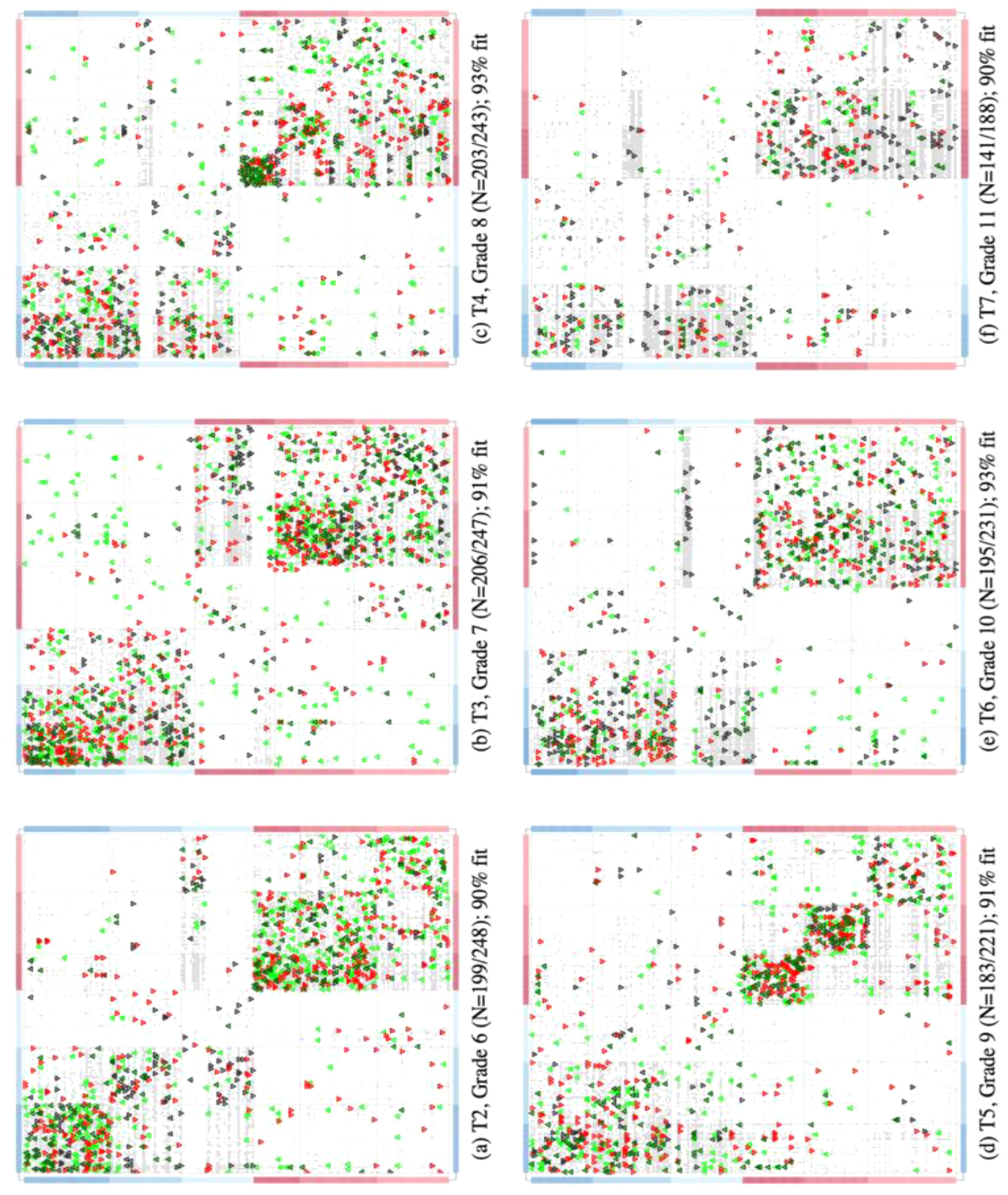
Fitness topology and relational niches, example (cohort: CM116C2). Note: The state of reported relations is encoded as follows: dormant ties are identified by dark gray squares; superimposed triangles identify the status of reported ties. Dark green triangles identify stable relations that are reported at both the current measurement and the next. Light green triangles identify relations that will first be reported at the next measurement. Inverted triangles identify relations that will not be reported again at the next measurement; either because the respondent did not list them as friend anymore (hollow, red) or because the respondent did not participate at the next measurement (hollow, gray). Panel labels include the number of participants and cohort size (in brackets) and the share of reported ties that are fit ties.

To establish the scope for viable relational dynamics, we consider how dormant ties are distributed within a setting. To this end, we sort individuals into groups with the aim of identifying promising sites for relational activity. Fig. 5 overlays the activation potential of ties onto a representation of the networks’ adjacency matrices of one setting studied, CM116C2. Fit ties (*f* ≥ .83) are identified as light gray squares and coloured symbols represent the status of reported relations for each corresponding matrix element. A blockmodeling algorithm has been applied to order individual on either axes so that fit ties cluster together.^5^

The coloring of the border of each panel encodes self-reported sex as the primary marker of social identity of the individual whose outgoing ties are represented by the corresponding row of the adjacency matrix (with incoming ties represented by the corresponding column). Stable relations that will be reported again are identified by dark green triangles; light green triangles identify relations that will first be reported at the next measurement. Relations that will not be reported again are identified by inverted red triangles. To correct for attrition, we only visualize individuals who participated in the study at that measure and the next (the fitness metric is based on the complete network, however).

Fig. 5 highlights that dormant ties cluster, suggesting that subsegments of the population differ in their propensity to engage in interactions and other relational activities. We refer to the intersection of subsegments as ‘relational niches’ and consider the prevalence of dormant ties as indicators of a niche’s short-term potential to sustain social relations. Dormant ties are a resource base for lower-level variation in relational activity which may (but need not) conform to the relational proscriptions of the setting. To the extent that lower-level entities pursue diverse relational goals, relational niches can sustain a more variegated network composition than the homogenous micro-mechanisms that population-level models account for. In turn, this suggests an answer to the puzzle of macro-level variation emerging from the homogenous micro-level relational norms that was formulated in the introduction. Observation 5 develops this point in a principled manner.

#### Observation 5: the ambiguity of dormant ties

Observation 4 introduced the idea that dormant ties establish a resource base for viable interactions and relational activity. Interactions and relations that activate dormant ties are aligned with the setting and therefore less likely to be selected against than interactions involving unfit dyads; from an ecological perspective, dormant ties are foregone relations. In the short run, any realized set of ties is thus merely one well-fitting configuration drawn from a larger set of viable configurations at that time and in that setting. The ecology itself does not favor any one of these sets over the other. Within limits, clusters of dormant ties would therefore sustain relational activities that transgress the relational norms of the setting. Observation 5 considers the extent to which members of the population can exploit dormant ties to pursue relational activities that contravene the proscriptions of top-down constraint.

Overall, it seems phenomenologically unlikely that all individuals in a population will choose their relations by the same exacting criteria of the setting. Yet this assumption is trivially implied by the assumption that model estimates derived at the population level adequately represent individual behavior. To relax this assumption, we simulated relational dynamics on the fitness topologies of the 252 networks in our sample while varying individuals’ underlying motivations to form relations with others. Each simulation run, every student (=agent) in each network is randomly assigned to one of five social identities with distinct relational preferences. As we detail below, some individuals seek to affiliate with popular peers, others value preexisting connections through mutual acquaintances. Agents are seeded with a capacity to interact with nine peers, make relational overtures to six, and accept nine incoming friendships. At each iteration, each agent evaluates their current interactions against the expected benefit of switching to a randomly matched alternative partner. These pairwise comparisons are based on ego’s assigned relational profile, as is ego’s subsequent identification of the six highest-valued interactions, to whom ego makes relational overtures. If accepted, these overtures become relations that are subjected to the ecological pressures of the setting: fit relations are preserved while unfit relations (*f* < .83) are eliminated.

Each of the five social identities is given a label that summarizes their underlying relational profiles: *Conformists* adhere to the norms of the setting that they find themselves in. Recall from Observations 1 and 2 that all 252 analyzed networks turned on positive relational norms on popularity (status), mutual acquaintances (clustering), and reciprocity. This is also how conformists value their own interactions with others, decide whom to make relational overtures to, and which incoming relational overtures to accept. Conformists form a baseline against which to compare the relational outcomes of other subgroups. *Status-seekers* strive for social standing. Their relational profile is to befriend those with many friends and to prefer being befriended by those with many friends. In contrast, *group-oriented* individuals value being part of an egalitarian community. Their relational profile is to seek out mutual acquaintances while avoiding popular peers. *Individualists* reject being part of a larger social group. Their relational profile is antithetical to the setting in the sense that they prefer to connect with individuals who are neither popular nor who belong to dense cliques. *Intrepids* transgress established categorization norms. Their relational profile is aligned with the setting, but they seek to befriend members of the other sex. As we did not adjust individuals’ reciprocity-preferences, agents always prefer connecting to peers who reciprocate their relational overtures (over connecting to peers who do not).

The simulation proceeds as follows: Each run is seeded on one of the 252 reported networks. At initialization of each run, every student in the setting (hereafter: agent) is randomly assigned to one of the five aforementioned identities. For that run, these identities define the ‘value’ that ego assigns to connecting to any of the other members of the population given the network of expressed relations. *Status-seekers*, for example, prefer to connect to peers who have many friends, *group-oriented* individuals value mutual acquaintances, and *intrepids* prefer affiliating with members of the other sex. At initialization, each agent is paired with nine interaction partners selected at random from the population. Then, relational dynamics proceed in three stages. First, ego compares the value of repeating each of their nine interactions against that of switching to an alternative tie, randomly drawn from the population. Second, ego makes relational overtures to the six interaction partners who score highest on ego’s valuation. A relation forms if that overture is accepted by its intended recipient.^6^ Finally, misaligned relations, i.e., with *f* < .83 (cf. Observations 1–3), are selected against and eliminated.

**Fig. 6.**
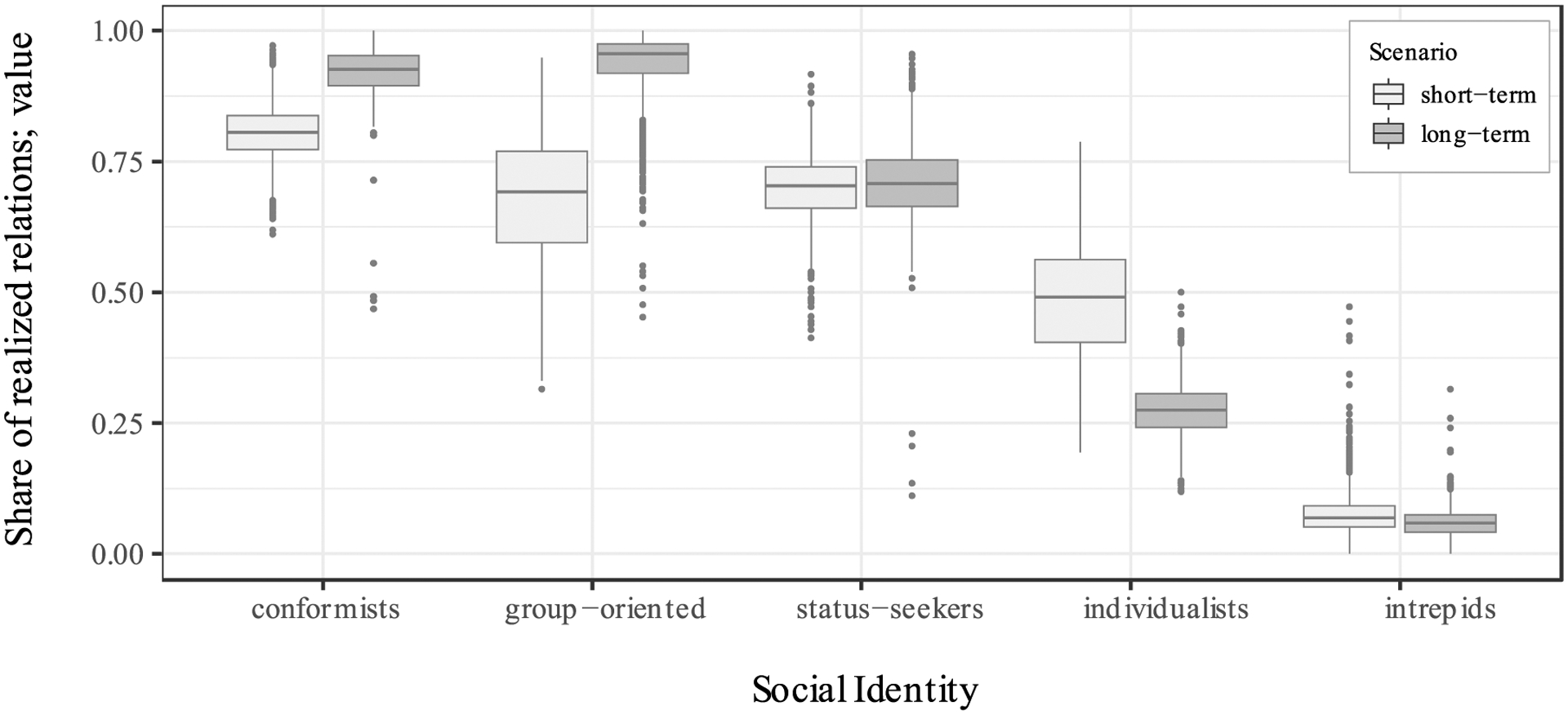
Simulation output: Share of realized relations by simulated social identity. Note: The boxplots summarize the share of relations realized (relative to simulated capacity) for the 5 social identities at the 100th iteration of 10 simulation runs seeded on the 252 networks. Two scenarios are considered: a short-run scenario with relational niches fixed at initialization and a long-run scenario where niches evolve over time. The boxplots thus summarize 5×10×252 × 2 = 25,200 observations.

For each of the 252 networks, we simulated two scenarios: one in which fitness is held fixed on the reported network (at initialization), and one in which fitness is updated as relational configurations evolve at each iteration (in both scenarios, the coefficients of the fitness function are fixed). The first scenario models the short-term constraint that a setting imposes on divergent relational interests; the second considers a long-term equilibrium for the setting given the demographic composition of the population. Fig. 6 reports the average share of realized relations (relative to the simulated capacity) for each identity group after 100 iterations for 10 runs each across the 252 networks analyzed.

Fig. 6 reveals that aligning one’s relational activities with the demands of a setting is an evolutionarily adaptive strategy in both the short- and the long-term: The highest proportion of relations in the population is observed among *conformists*, who realize 80.5% of their relational capacity on average in the short-run and 91.3% on average in the long-run. As their profiles value ties that fit the setting, conformists have only few of their relational overtures selected against.

Remarkably, several of the social identities with divergent relational profiles are also able to maintain a substantial number of relations despite contravening the setting’s relational norms. Even though their ties are selected against more frequently than those of conformists, *group-oriented* individuals on average realize 63.9% of their capacity in the short-term while *status-seekers* realize 70.5%. In the long run, clustering even increases relational stability: members of group-oriented identities overtake conformists and increase their share of realized relations to 91.4%. Even identities whose relational profiles are antithetical to the setting, such as *individualists* who avoid groups and reject status-seeking, realize 45.3% of their capacity on average in the short-run, and 27.8% in the long-run. Only *intrepids*, who transgress prevalent categorization norms, fail mostly to realize their preferred relations (here: with the other sex). Extending on Observation 4, which introduced the concept of relational niches, Observation 5 thus suggests that individuals with norm-divergent relational profiles can – within limits – leverage dormant ties to pursue their respective goals.

In the introduction, we noted a basic puzzle concerning social networks: the emergence of different macro-level topologies from the same relational micro-mechanisms. Having applied the framework presented in the first part of this paper to the illustrative case of adolescent friendship, we can attempt to address this puzzle given the insights generated by Observation 5: it is that social networks retain a quantifiable scope for tie variation, bounded as relational niches, that allows members of a population to pursue idiosyncratic and possibly norm-divergent relational activities. This scope for variation is a direct consequence of differences in the timescales at which the various elements of the relational system coevolve: by definition, higher levels are too inert to map onto and perfectly control lower-level dynamics. The resultant scope for tie variation allows lower-level entities to pursue relations that are only moderately aligned with the proscriptions of the setting. Within specifiable limits, two settings with identical relational norms can therefore nonetheless sustain disparate network topologies that exploit viable lower-level variation in relational configurations.

## Discussion

In the first part of this paper, we presented an ecological framework to model, explain, and predict the coevolution of social networks with their proximal environments. Drawing on the two tenets of (i) tie variation and selective retention and (ii) hierarchical ordering of contexts via timescale separation, we introduced tie fitness as a time-varying predictor for the longevity of social relations. Drawing on longitudinal network data collected from 36 cohorts of North American high school students, we then presented five observations regarding the ecology of adolescent friendship. Whereas the first three observations established ‘tie fitness’ as a metric on which to base subsequent analyses, observations four and five identified dormant ties as a resource base allowing members of the population to pursue their idiosyncratic relational goals. Clusters of dormant ties channel relational variation in ways that allow for the emergence of different network structures from the same relational mechanisms.

Network ecology as presented here introduces a systems component to the study of social network dynamics. Individuals’ efforts to interact and maintain social relations with others play out within broader systems of relational norms and expectations. Relational dynamics unfold at a meso-level timescale, above that of fleeting interactions and below that of relational norms and expectations that tie to relatively stable social identities. This timescale-separated hierarchy implies a temporally mediated connection between different levels of contexts: ties grow more durable, become more information-laden, and change more slowly as one moves up from interaction to dyadic relation to group affiliation and social identity. These differences in inertia imply that higher levels constrain lower levels of the relational system in the short run: social identities constrain relations, and relations constrain interactions. At the same time, however, their inertia also implies that higher levels cannot map directly onto lower levels and therefore cannot perfectly control lower-level activities. Therefore, each level of a timescale-separated system retains some scope for variation that entities at lower levels can exploit to increase their representation at the next-higher level. In turn, this suggests that social networks will be more adaptable, pliable, and open to interpretation than a naïve structuralist approach might suggest.

The recognition that higher-level constraint must generalize across lower-level variation and thereby allow for a quantifiable scope for lower-level variation is a universal feature of complex adaptive systems. This insight opens the presented network-ecological framework to a more general account of network dynamics and applications beyond the illustrated case of adolescent friendship. At this general level, the shift in emphasis from expressed relational configuration to modeling the scope for tie variation (as the activation potential of fit ties) turns attention to the social processes that channel tie variation in social settings. We leave a deeper examination of these processes for future research. In lieu of a conclusion, we suggest three lines for further elaboration: classifying and modeling drivers of social selection and the strictness of ecological constraint, network design and the coevolution of relational norms with their proximal environments, and the changing salience of social identities over time.

This first line of enquiry centers on the ecological framework’s application to different use cases. Modeling relational dynamics in terms of the network ecological framework requires identifying the relevant drivers of social selection and parameterizing a well-specified fitness function accordingly. As our illustrative use-case was aimed at identifying broad themes of the ecology of adolescent friendship, we here opted for a relatively simple specification; considering only self-reported sex, ethnicity, and parental socioeconomic status alongside indegree popularity, cohesion, and reciprocity as factors influencing adolescent friendship. Other use-cases will call for more detailed specifications and more elaborate statistical models to capture relational interdependencies. For example, the fitness function must be adjusted before it can be applied to other settings (e.g., schools in Asia or Europe), domains (interfirm alliances in industries), or types of social relations (e. g., advice-seeking, romance, or rivalry).

On a related note, identifying dormant ties (i.e., the resource base for tie variation) implies some understanding of the strictness of ecological constraint, as operationalized by the fitness threshold. Some settings, such as chains of command in the military or hierarchies in formal bureaucracy, are heavily constrained and assign fixed roles to individuals, limiting interactions to align with strict relational norms (Martin, 2009). In such contexts, interactions closely align with interlocking roles and generalized relational expectations; social actors know what is expected of them. Other contexts are more open and undefined, entailing ambiguity and multiple opportunities for experimentation (e.g., school outings, or *Mardi Gras*). In either case, the strictness of constraint is defined by the threshold used to identify fit ties. In strict settings, the threshold will be high, limiting the resource base for norm-deviant relational activities; in lenient settings, the threshold will be lower, allowing for more dormant ties and diminished predictability of ensuant relational dynamics. By developing a principled understanding of tie fitness alongside the strictness with which settings enforce selection pressures, network ecology holds the promise of both predicting and explaining the coevolution of relational dynamics within diverse social settings.

The second line of enquiry is to consider criteria for effective design of social settings. For networks to stabilize, different levels of contexts must accomplish a degree of continuity that is often engrained in purposive design. For example, organizations assign occupational titles to their employees and formulate job descriptions that encode role expectations and generalized communication paths and subordination relations for employees in ways that constrain relational mechanisms over time, “forcing” individuals to reproduce both the contexts and the configurations in which they form relations with others (e.g., Bourdieu, 2005). Schools require students to collaborate on activities based on class enrolment and curriculum, and country-specific legislations regulate corporate alliances and interorganizational networks. To a substantial degree, the relational configurations that are studied as social networks thus depend on the respective contexts in which they occur. This calls for domain-specific explanations for tie formation, longevity, and dissolution of different types of social relations in diverse social contexts.

Finally, a third line of enquiry considers the salience of social identities and their part in shaping the coevolution of networks and their outcomes. Particularly in dynamic environments in which social configurations evolve rapidly and identities are unclear, social actors may leverage their network positions to shape prevalent relational norms in their favor. In this broader context, social identities suppose an understanding of group membership (who is part of an identity and who is not?) and definitions of role-expectations that are partly open to interpretation and subject to modification. Markers of social identity and their (time-varying) significance for relational dynamics are discursively negotiated to build identities around conceptions of skin tone and ethnicity, occupational roles, gender roles, or class habitus. This calls for domain- and specific relational explanations for the emergence, stabilization, and possibly decline, of different types, or species, of social identities and their attendant relational profiles. Going forward, a task of network ecology is to uncover social processes in which these varied relational dynamics coevolve - at distinct levels of contexts, across settings, and over time.

## Figures and Tables

**Table 1:** Regression coefficients establishing relational norms for setting CM116C2.

	*Dependent variable: Relation (0/1)*						
	(T1)	(T2)	(T3)	(T4)	(T5)	(T6)	(T7)
*Categorization norms: (Homophily on* …*)*							
Self-reported sex (m/f)	2.139	1.311	1.536	1.488	0.871	1.625	1.436
Ethnicity (maj./min.)	0.181	0.421	0.25	0.36	−0.128	0.066	0.199
Parental SES (high/low)	0.265	0.345	0.097	0.169	0.134	0.095	0.266
*Configurational norms*							
Reciprocity	2.909	2.611	2.881	2.934	2.945	3.645	2.909
Status-orientation	0.573	0.459	0.539	0.588	0.47	0.593	0.573
Local clustering	0.523	0.733	0.768	0.863	0.742	0.648	0.523
Constant	−7.827	−7.211	−7.145	−7.609	−6.414	−7.344	−7.234
Observations	57,360	61,256	60,762	58,806	48,620	53,130	35,156
Log Likelihood	−2254	−2756	−2789	−2383	−2037	−1927	−1341
Akaike Inf. Crit.	4525	5531	5597	4784	4092	3872	2699
Bayesian Inf. Crit.	4606	5612	5678	4865	4171	3952	2775

Note: SES = socioeconomic status, derived from students’ eligibility for school lunch program. Ethnicity is binary-coded as belonging either to the largest ethnicity in the setting (majority), or to any minority. Standard errors omitted.

**Table 2:** Tie assortativity by categorization norms.

Time	# of Settings	Parental SES (high/low)			Ethnicity (majority/minority)			Self-reported sex (boy/girl)		
		av	min	max	av	min	max	av	min	max
T1	36	0.13	−0.03	0.32	0.07	−0.08	**0.55**	**0.87**	**0.76**	**0.94**
T2	36	0.14	−0.04	0.33	0.08	−0.05	**0.55**	**0.84**	**0.72**	**0.93**
T3	36	0.14	−0.04	0.35	0.11	−0.05	**0.58**	**0.82**	**0.69**	**0.92**
T4	36	0.14	0.01	0.27	0.13	−0.05	**0.56**	**0.71**	**0.56**	**0.83**
T5	36	0.15	0.02	0.24	0.15	−0.04	**0.48**	**0.70**	**0.56**	**0.84**
T6	36	0.12	−0.07	0.30	0.10	−0.08	**0.49**	**0.67**	**0.49**	**0.81**
T7	36	0.13	−0.03	0.34	0.10	−0.06	0.43	**0.65**	**0.46**	**0.86**

Note: At each measurement (T1–T7), averages and extrema are reported for the 36 settings. Parental Socioeconomic status (SES) is approximated by eligibility for the school’s subsidized lunch system. Ethnicity is binary-coded as an individual belonging either to the largest ethnicity represented in the setting (majority), or to any ethnicity minority. Coefficients greater than 0.45 are emphasized in bold.

**Table 3:** Fitness predicts friendship reporting, formation, longevity, and survival.

	Dependent Variables:			
	is reported	will form	longevity	survival
	(0/1)	(0/1)	(count)	(0/1)
	M3a	M3b	M3c	M3d
*Ecological Fitness*				
Dyad Fitness [0,1]	18.030 * **	10.372 * **	3.770 * **	7.438 * **
	(0.120)	(0.078)	(0.055)	(0.193)
*Quality of relations*				
Times reported in past			0.125 * **	0.224 * **
			(0.003)	(0.006)
Is ‘best friend’			0.367 * **	0.938 * **
			(0.008)	(0.014)
Meet 1–2 times a month			0.157 * **	0.225 * **
			(0.012)	(0.020)
Meet once a week			0.218 * **	0.328 * **
			(0.013)	(0.023)
Meet a few times a week			0.213 * **	0.379 * **
			(0.013)	(0.022)
Meet almost every day			0.179 * **	0.362 * **
			(0.013)	(0.022)
*Measurement*				
T2	0.132 * **	0.252 * **	−0.362 * **	−0.871 * **
	(0.009)	(0.015)	(0.012)	(0.023)
T3	0.145 * **	0.272 * **	−0.508 * **	−0.840 * **
	(0.010)	(0.014)	(0.012)	(0.023)
T4	0.109 * **	0.039 * *	−0.713 * **	−0.894 * **
	(0.011)	(0.015)	(0.013)	(0.024)
T5	−0.001	−0.149 * **	−0.923 * **	−0.898 * **
	(0.011)	(0.016)	(0.014)	(0.026)
T6	−0.051 * **	−0.220 * **	−1.280 * **	−0.900 * **
	(0.012)	(0.018)	(0.017)	(0.029)
T7	−0.085 * **	−0.294 * **	−1.917 * **	−1.098 * **
	(0.014)	(0.020)	(0.022)	(0.033)
Constant	−17.732 * **	−11.387 * **	−2.828 * **	−6.883 * **
	(0.116)	(0.078)	(0.057)	(0.188)
Observations	5311,374	5197,680	113,694	113,694
Groups (dyads)	1795,501	1784,763	68,596	68,596
Log pseudolikelihood	−340,053	−238,220	−165,991	−67,769
Wald chi^^^2	42,189	31,518	18,701	11,701
Pseudo-R^^^2	0.3812	0.2406	0.0636	0.1323

Note: All analyses are of dyads reported by individuals who participated in both the current and the next measurement. All models control for school setting (omitted to preserve space). Robust standard errors in parentheses, clustered by dyad. p < 0.01, p < 0.05, p < 0.1

## Footnotes

1 In adopting Darwinian vocabulary, we do not suggest reducing social phenomena to biophysical processes. Nor do we intend to rehash old debates about importing concepts from biology into the social sciences by naïve analogy (Degler, 1991; Runciman, 1998). Instead, our aim is to consider how the three generalized principles of variation, selection, and retention can be used productively to study network dynamics (for broader calls to generalize Darwinism, see, e.g., Aldrich et al., 2008; Bickhard and Campbell, 2003; Hodgson and Knudsen, 2010). In short, we want to develop network ecology as a novel perspective on the etiology, morphology, and adaptiveness of social relations to evolving contexts.

2 At a deep level, what counts as appropriate in a setting is socially and relationally construed; as is theorized, e.g., by the concept of ‘appropriateness judgements’(Lazega, 2014). However, the ecological view remains agnostic about the normative bases of appropriateness judgments. Instead, group- and population level patterns guide and constrain lower-level relational activities that, in turn, accumulate to group- and population level patterns. Strictly, the ecological framework therefore does not require knowledgeable agents who are aware of the (im-)propriety of a tie: extant relational norms are enacted even if individuals do not fully comprehend the local context, culture, or rituals that they are grounded in (Bearman, 1997).

3 Students self-reported their sex in a forced-binary survey item (male/female). The database includes responses that have been harmonized across measurements. Thereby, an unambiguous sex has been assigned to 97.6% of all students in the population (N = 8907), accounting for 99.1% of all individuals surveyed across all eight waves (N = 43,343). While this approach maximizes data completeness, as one reviewer has correctly pointed out, the chosen design precludes the possibility of students identifying as non-binary, a self-categorization that contemporary societal discourse links strongly to social identity. Relatedly, non-response to this item, or inconsistent responses over time, might also reflect self-expressions of social identity. Given the design of the dataset, we cannot consider these aspects in our analysis. We do note, however, that only 25 of the 214 students without an assigned sex completed one or more surveys in the first place.

4 Another way of thinking about fitness is in terms of the costs of maintaining a relation. In many cases, two individuals may maintain a tie that violates the relational norms of a setting, but in doing so, they incur a cost that is inversely proportional to the fitness of their ties. This extension seems particularly relevant in the context of individuals’ efforts to mobilize ties to effect cross-level changes (Lazega, 2016).

5 As our aim is to identify clusters of fit ties, we grouped individuals based on (dis-)similarities in the fitness of their outgoing and incoming active or dormant ties to other members of the population. Specifically, we used hierarchical clustering (Ward, 1963) to group individuals based on the covariation of fitness of their incoming and outgoing ties to the other.

6 As simplifying assumption, agents have capacity to maintain nine incoming friendship relations. If they receive more overtures than that, they accept those overtures which best match their own relational preferences. We assume that relational preferences are symmetrical: an agent with a preference to befriend popular peers prefers to be befriended by popular peers; an agent with a preference to befriend alters with mutual acquaintances prefers to be befriended by alters with mutual acquaintances.
